# The Biosorption Capacity of the Marine Microalga *Phaeodactylum tricornutum* for the Removal of Toluidine Blue from Seawater

**DOI:** 10.3390/toxics12040277

**Published:** 2024-04-09

**Authors:** David Fernández, Julio Abalde, Enrique Torres

**Affiliations:** Laboratorio de Microbiología, Facultad de Ciencias, Universidade da Coruña, Campus de A Zapateira, 15071 A Coruña, Spain; davidfdz182@gmail.com (D.F.L.); abaldej@udc.es (J.A.A.)

**Keywords:** microalgae, toluidine blue, biosorption, seawater, bioremediation, living biomass

## Abstract

A wide variety of dyes, such as toluidine blue (TB), are used daily for a multitude of purposes. After use, many of these compounds end up in aqueous effluents, reaching natural environments, including marine environments. The removal of these pollutants from marine environments must be considered a priority problem. The search for natural techniques, such as biosorption, is a preferred option to eliminate pollution from natural environments. However, biosorption studies in seawater are scarce. For this reason, the living biomass of the marine microalga *Phaeodactylum tricornutum* was studied to determine its ability to remove TB from seawater. The kinetics of the biosorption process, the isotherms, and the effect of light and pH were determined. This biomass showed a maximum TB removal capacity of 45 ± 2 mg g^−1^ in the presence of light. Light had a positive effect on the TB removal capacity of this living biomass. The best fitting kinetics was the pseudo-second order kinetics. The efficiency of the removal process increased with increasing pH. This removal was more effective at alkaline pH values. The results demonstrated the efficacy of *P. tricornutum* living biomass for the efficient removal of toluidine blue dye from seawater both in the presence and absence of light.

## 1. Introduction

There are more than 100,000 commercially available dyes, with an annual production of 7 × 10^5^ tons [1]. Currently, dyes have multiple applications, with the textile industry being the largest user of these compounds [2]. About 2–20% of the used dyes are directly discharged into aqueous effluents, and given the good solubility of these compounds, they can be found in significant quantities in polluted media [3]. Although dyes can be found in low concentrations in water bodies, they become highly visible [1], even modifying the perception of the landscape. Furthermore, dyes are able to block sunlight, which is essential for many photoinitiated chemical reactions, inducing photoinhibition in light-dependent aquatic organisms [1,4]. This problem results in a loss of diversity in ecosystems [5].

An added problem is that the chemical structure of dyes makes them resistant to degradation and discoloration upon exposure to light, water, and a wide range of chemicals [6], which turns the dyes into recalcitrant compounds [7], presenting a high capacity for persistence in the environment and an ease of mobilization and accumulation in sediments [8]. These characteristics constitute threats to both human health and wildlife [9].

Toluidine blue (TB) is an acidophilic metachromatic dye that stains acidic components of tissues (sulphates, carboxylates, and phosphate radicals) and is soluble in both water and alcohol. It is a dye that is mainly used in the medical field, textile industry, and biotechnology, as a mediator of several reactions such as photosensitizer and identification of organisms [10], which makes this dye a potential waste in educational and industrial institutions. As a consequence of these uses, this dye can reach aquatic environments causing a pollution problem. Some studies are looking for efficient techniques to remove TB from the environment, using gypsums [3], photocatalysis [11], or the synthesis of nanomaterials [12], which indicates the importance of using effective remedies to reduce the concentration of TB in the environment. It is important to highlight that pollution in marine environments has accelerated in recent centuries due to the number of discharges that reach the sea. However, studies to remove pollutants from seawater are scarce [13,14].

The search for plausible solutions that can be applied in situ is of great importance today. These solutions must demonstrate their efficiency, taking into account the environmental conditions of the place where these solutions need to be applied. Bioremediation is an excellent option to mitigate the adverse effects of pollutants on the environment through the removal or metabolization of toxic substances [15]. Microorganisms (bacteria or microalgae), fungi, or plants can be used to remove pollutants from water and soil [16]. These bioremediation techniques are more affordable, environmentally friendly, and produce less waste after use [17]. For these reasons, it would also be desirable to be able to apply this technique to eliminate pollutants from seawater. However, biosorption studies of pollutants in seawater are also scarce [18,19,20]. Most experiments use deionized water where the assayed pollutant is dissolved. However, using seawater in the experiments is a necessary step to appropriately assess the characteristics of a specific biosorbent intended to eliminate pollutants from the marine environment.

Biomass derived from microalgae is currently considered a good candidate to remove harmful substance from the environment [21]. Microalgae can remove pollutants through biosorption, bioaccumulation, or biodegradation processes [15], and these microorganisms can even use pollutants as nutrients and degrade them enzymatically [5]. The carbohydrates present in the cell walls of microalgae make them capable of adsorbing large amounts of toxic chemical compounds such as heavy metals, phosphorus, nitrogen, and other complex compounds from water bodies [15]. In addition, these microorganisms offer other advantages; for example, microalgae can easily capture the CO_2_ gas due to their photosynthesizing ability and elaborate by-products such as lipids, vitamins, and carbohydrates [22], considering that CO_2_ biofixation is one of the most successful alternatives to remove CO_2_ from the atmosphere [23]. Microalgae are fast-growing due to their simple structure [5,24], which facilitates their cultivation and growth.

*Phaeodactylum tricornutum* is a marine microalga that belongs to the group of diatoms and whose biomass has been shown to have good characteristics to remove pollutants [19,25]. This microalga’s remarkable properties allow it to be used in processes based on biosorption techniques. In addition, it is an easy-to-cultivate microalga, widely used in laboratory tests and in aquaculture; this makes the biomass of this microalga easily available. For these reasons, the main objective of this study is to determine the capacity of the *Phaeodactylum tricornutum* living biomass to remove TB from seawater. The kinetics of the process, the equilibrium isotherms, and the effect of light and changes of the pH in the medium on the removal capacity will be studied.

## 2. Materials and Methods

### 2.1. Stock Culture of the Microalga

*Phaeodactylum tricornutum* was grown in 1 L Pyrex glass bottles in a culture chamber at a temperature of 18 ± 2 °C with 12/12 h light/dark cycles and a light intensity of 68 µE m^−2^ s^−1^. Natural seawater, previously autoclaved at 121 °C for 20 min and enriched with ALGAL culture medium, was used. The culture was blown with filtered air (0.22 µm pore size) at a flow rate of 10 L min^−1^.

### 2.2. Seawater

The seawater used in the experiments was natural seawater, filtered (0.22 µm pore size) and autoclaved at 121 °C for 20 min. This seawater was collected in the bay of La Coruña (N43°22′21.5116″ W8°24′32.6953″). Salinity was 35‰, and pH was 8.2 ± 0.1, adjusted with HCl or NaOH when necessary.

### 2.3. Toluidine Blue Dye

The dye used in the experiments was Toluidine blue (TB) (CAS = 92-31-9; CI = 52040). It is a cationic dye belonging to the thiazine group and its chemical structure is C_15_H_16_CIN_3_S. This dye was obtained from Merck (Rahway, NJ, USA).

A stock solution of the dye was prepared in deionized water at a concentration of 1 g L^−1^.

### 2.4. Biosorption Assays

The biosorption experiments were carried out in 50 mL Kimax tubes with different TB concentrations. The amount of biomass used in the experiments was equivalent to 0.4 g L^−1^ of dry biomass. This biomass was obtained from the stock culture of the microalga. To determine the volume that was necessary to take from this stock culture, the cell density of the culture was determined using a Neubauer counting chamber. These data, together with the value of the dry weight of the microalga, allowed the volume needed to obtain the equivalent amount of dry biomass to be determined. The volume obtained from the stock culture of the microalga was centrifuged at 1700× *g* × 3 min to obtain the microalgal biomass that was transferred to the Kimax tubes. Seawater and a volume of the stock dye solution were added to these tubes to obtain the dye concentrations tested of 1, 2, 4, 8, 10, 20, 40, 60, 80, and 100 mg L^−1^. The tubes were incubated in the culture chamber at 18 ± 2 °C, with shaking at 200 rpm and a light intensity of 68 µE m^−2^ s^−1^.

Control tubes exposed to light and in the dark (covered with aluminum foil) without biomass were used to determine the stability of the dye in seawater, and since the experiments were performed with light, it was also determined whether the dye underwent photodegradation.

For all experiments, samples were taken at time intervals of 1, 5, and 10 min, and 0.25, 0.5, 1, 2, 3, and 4 h. The samples were centrifuged at 6000× *g* × 2 min, and the supernatant was transferred to a −20 °C freezer for subsequent measurement in a spectrophotometer.

#### 2.4.1. Effect of Light on the Biosorption Process by the Living Microalga

To study the possible effect that light may have on the biosorption process of the dye by the living cells of the microalga, experiments were carried out with tubes containing microalgal biomass identical to those indicated above but covered with aluminum foil. The tubes were sampled in the same way as described above.

#### 2.4.2. Determination of the Effect of pH on Biosorption Capacity

To determine the effect of pH on the biosorption capacity of this dye by the living cells of the microalga, seawater at different pH values was used, obtained by adjustment with HCl or NaOH depending on the pH. The pH values tested were 2, 4, 6, 8, and 10. The assays were similar to those indicated above, but with a single dye concentration of 20 mg L^−1^. Control tubes without biomass were also used to determine the stability of the dye at different pHs. Aliquots were taken at 1, 5, and 10 min, and 0.25, 0.5, 1, 2, 3, and 4 h, and then stored in a −20 °C freezer for subsequent measurement in the spectrophotometer.

### 2.5. Analytical Methods

The amount of dye removed in the experiments was determined spectrophotometrically at a wavelength of 626 nm. For this purpose, a calibration curve was carried out with different concentrations of the dye in seawater.

The amount of dye removed per unit of biomass was determined by:

(1) $$q_{t}=\frac{\left(C_{i}-C_{t}\right)*V}{m}$$

where *q_t_* (mg g^−1^) is the amount of dye removed per unit of biomass at time *t* (h), *C_i_* is the initial dye concentration in the control tubes (mg L^−1^), *C_t_* is the dye concentration (mg L^−1^) of the samples with microalgal biomass at time *t*, *V* is the volume of the solution (L), and *m* is the mass of the sorbent (g).

The percentage of dye removed was determined by:

(2) $$P_{t}=\frac{\left(C_{i}-C_{t}\right)*100}{C_{i}}$$

where *P_t_* is the percentage at time *t*.

#### Analysis of Kinetics and Equilibrium Isotherms

To determine the properties of *P. tricornutum* biomass as a TB biosorbent, the kinetics of the process were studied, both over time and at equilibrium (isotherms). The data obtained were fitted to several kinetic and isotherm models (Table 1).

### 2.6. Determination of the Zero Charge Point of the Biomass

The zero charge point (pH_ZCP_) is the pH value at which the charge of the biomass is zero (equal number of positive and negative charges). This value is important when performing biosorption studies with charged compounds such as this dye. The charge of the biomass surface is an important parameter to optimize the biosorption process. To determine the pH_ZCP_, biomass samples of 0.4 g L^−1^ were washed with a 0.6 M NaCl solution and collected by means of centrifugation at 6000× *g* × 2 min. The biomass samples thus obtained were diluted in 0.6 M NaCl solutions but with different pHs (2–13) (pH_i_) in 50 mL tubes. These solutions were allowed to equilibrate for 4 h. After this time, they were centrifuged at 6000× *g* × 2 min, and the pH of the supernatant of each of these solutions was measured (pH_f_). The pH_ZCP_ is obtained from a plot of ΔpH (pH_f_ − pH_i_) versus pH_i_.

### 2.7. Statistical Analysis

All assays were conducted in triplicate. Data are the mean of the three replicates ± SD. The fits to the kinetic and isotherm models were performed by means of non-linear regression. The coefficient *r_adj_*^2^ was used as a measure of goodness of fit. To establish whether there were differences between the results of the experiments with the different factors, a two-way ANOVA and Turkey’s test were used, establishing a significance level of α = 0.05. Previously, it was verified that the ANOVA requirements were met.

## 3. Results

### 3.1. Study of the Stability of the TB Dye in the Experimental Conditions

Control experiments (tubes exposed to light and tubes in darkness) were carried out without the presence of microalgal biomass to determine the stability of the dye in seawater, and the possible photodegradation effect that may occur on the dye since the experiments were carried out with illumination to keep the biomass alive. The results obtained from the assays in seawater and from the tubes in darkness, and analyzed using a two-way ANOVA (factors: time (initial and final of the assays), and initial dye concentration) indicated that there were no significant differences between the initial and final dye concentration (F_1,40_ = 0.40; *p* = 0.53) for all the dye concentrations tested. Therefore, the dye remained stable in seawater during the 4 h of the assay. Similarly, the tubes of these control experiments exposed to light, also in seawater, allowed the determination of a possible photodegradation of the dye during the biosorption process. The results obtained and analyzed by means of a two-way ANOVA (factors: time (initial and final of the assays), and initial dye concentration) indicated that there was no significant variation between the initial and final concentration of the dye at all the concentrations tested (F_1,40_ = 1.61; *p* = 0.21); therefore, there was no photodegradation of the dye during the assays. Therefore, the TB dye showed stability in seawater and no photodegradation under the microalga culture conditions during the 4 h of the process.

### 3.2. Effect of Microalgal Biomass on TB Dye Removal in the Presence of Light

**Figure 1:**
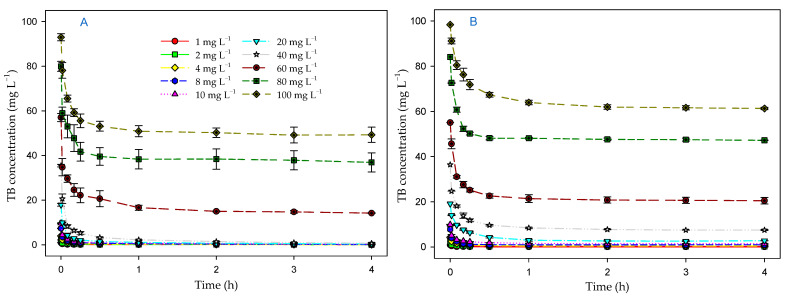
Evolution over time of the concentration of the dye in the medium (mg L^−1^) using the biomass of *P. tricornutum*, both in the presence of light (**A**) and in the dark (**B**).

Figure 1A shows the evolution of the dye concentration (mg L^−1^) over time in the experiments with microalgal biomass and with illumination. A clear decrease in the initial dye concentration is observed for all tested concentrations. The results analyzed by means of a two-way ANOVA (factors: time (initial and final), and initial dye concentration) showed that this decrease was statistically significant (F_1,40_ = 2971.41; *p* < 0.001) at all assayed concentrations of the dye. Furthermore, there was a significant interaction between both factors (F_9,40_ = 249.82; *p* < 0.001). This interaction occurred at the lowest initial dye concentrations, up to the concentration of 40 mg L^−1^, which removed a higher amount of dye than expected. Therefore, the *P. tricornutum* biomass was able to remove TB dye from the medium at all concentrations tested. This figure also shows a rapid initial removal of the dye, mainly in the first hour, with a removal of more than 50% of TB at concentrations of 1–10 mg L^−1^. Subsequently, equilibrium was reached quickly at the lowest concentrations tested. In fact, at these low concentrations (1–40 mg L^−1^), between 98 and 100% of dye was removed at the end of the process. At the highest concentrations (60–100 mg L^−1^), equilibrium was also reached due to the saturation of the microalgal biomass by the TB dye. At the highest concentration (100 mg L^−1^), 47% of the dye added to the medium was removed.

**Figure 2:**
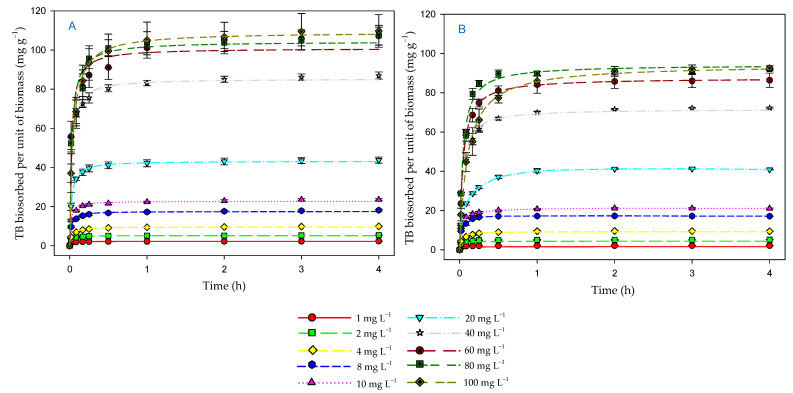
Evolution over time of the amount of dye biosorbed per unit of biomass (mg g^−1^), both in the presence of light (**A**) and in the dark (**B**).

Figure 2A shows the amount of dye removed per unit of biomass (mg g^−1^) in the presence of light with respect to time. It can be seen that the amount of dye removed by the biomass increased over time at all the dye concentrations tested. The results obtained by means of a two-way ANOVA (factors: time (initial and final), and initial dye concentration) showed that this increase was statistically significant (F_1,40_ = 4959.88; *p* < 0.001) at all the dye concentrations. The concentration of 100 mg L^−1^ was the one that presented the highest biosorption with 109.79 ± 10.35 mg g^−1^, compared to the concentration of 1 mg L^−1^ with 2.24 ± 0.02 mg g^−1^. This result shows that the *P. tricornutum* biomass was able to remove, although with different efficiency, the TB dye from the medium at all concentrations tested (1–100 mg L^−1^). A first phase of rapid removal can also be observed in this figure until equilibrium was reached.

### 3.3. Effect of Microalgal Biomass on Dye Removal in the Absence of Light

As occurred in the presence of light, the microalgal biomass removed the dye at all concentrations tested (Figure 1B), although with different degrees of efficiency depending on the initial concentration of the dye. After 4 h of process, the final dye concentrations in the medium were lower than the initial concentrations. The results analyzed by means of a two-way ANOVA (factors: time (initial and final) and initial dye concentration) showed that these differences were significant (F_1,40_ = 39,126.57; *p* < 0.001). There was also a significant interaction between both factors (F_9,40_ = 2729.03; *p* < 0.001), but in this case, only up to the concentration of 10 mg L^−1^. Therefore, the behavior was similar to the presence of light; however, the removal values obtained in darkness were lower than those obtained with illumination. When the results of both experiments were statistically compared by means of a two-way ANOVA (factors: light and final dye concentration), it was verified that this effect was significant at all the dye concentrations tested (F_1,40_ = 136.07; *p* < 0.001), which shows that there was an effect of light on the efficiency of the microalgal biomass to remove the dye. In addition, there was a significant interaction with the concentration factor (F_9,40_ = 20.30; *p* < 0.001). This interaction also occurred at lower dye concentrations, up to 40 mg L^−1^. In the dark, although there was a significant removal by the microalgal biomass, it was less efficient than in the presence of light. In fact, the dye was only entirely removed at concentrations of 1–2 mg L^−1^.

Figure 2B shows the amount of dye removed per unit of biomass in the absence of light. As in Figure 2A, it can be seen that, even in the absence of light, the amount of removed dye increased over time at all the concentrations tested. The results obtained by means of a two-way ANOVA (factors: time (initial and final), and dye biosorbed per unit of biomass) showed that this increase was statistically significant (F_1,40_ = 38,107.73; *p* < 0.001) at all concentrations. The concentration of 100 mg L^−1^ showed the highest biosorption with 94.43 ± 1.32 mg g^−1^, compared to the concentration of 1 mg L^−1^ with 2.13 ± 0.02 mg g^−1^. However, when compared to the amount removed in the presence of light, the amount removed was lower.

### 3.4. Effect of Contact Time and Initial Dye Concentration on Biosorption Process

Contact time is an important parameter to study the efficiency of a biosorption process. According to Figure 1, as the contact time increased, the dye concentration in the medium decreased. Initially, this decrease was rapid. In fact, most of the dye was removed during the first half hour. Gradually the decrease was slower until the equilibrium was reached. This equilibrium was reached at all concentrations tested during 4 h of the process. Therefore, this time was considered adequate to characterize the TB biosorption process by the microalgal biomass, both in the presence of light and in the dark.

**Figure 3:**
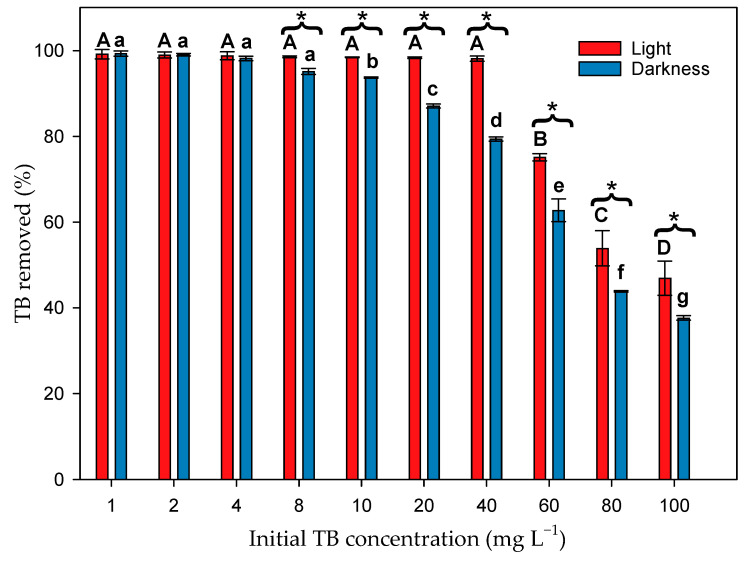
Percentage of TB removed by the microalgal biomass both in the presence of light and in the dark after 4 h. Different letters indicate significant differences (*p* < 0.05) between the initial concentrations of the dye for the cultures exposed to light (capital letters) and for cultures in darkness (lowercase letters). * Indicates significant differences (*p* < 0.05) between cultures exposed to light and cultures in darkness.

In the biosorption process, the initial concentration of sorbate influences the efficiency of the process. Figure 3 shows the percentage of TB removed by the microalgal biomass after 4 h. This percentage decreased as the initial TB concentration increased, decreasing the removal efficiency. However, there were significant differences between light and dark assays (F_1,40_ = 319.06; *p* < 0.001). There was also a significant interaction (F_9,40_ = 26.88; *p* < 0.001) between the light–dark factor and the initial dye concentration. In the presence of light, the interaction occurred up to a concentration of 40 mg L^−1^, while in the dark, only up to a concentration of 10 mg L^−1^. In the presence of light, 100% of the dye was removed at concentrations of 1–40 mg L^−1^. This percentage decreased to only 47% at the highest dye concentration tested. However, in the dark, although the trend was similar, 100% removal was only achieved at concentrations of 1–2 mg L^−1^, and with only 38% at the highest concentration.

### 3.5. Kinetic Models

Table 2 and Table 3 show the parameters obtained by fitting the data to the kinetics of the biosorption process using microalgal biomass, both in the presence (Table 2) and in the absence of light (Table 3). The kinetics are important to determine a model that can better explain the data on TB removal by microalgal biomass. Two kinetic models, pseudo-first order and pseudo-second order, were evaluated since they are the most commonly used models. Both in the presence and absence of light, and for all concentrations tested, the model that obtained the highest *r_adj_*^2^ was the pseudo-second order model. Comparing the data in both tables, the values obtained for the kinetic parameters in the presence of light were higher than the values obtained in the dark.

### 3.6. Biosorption Isotherms

**Figure 4:**
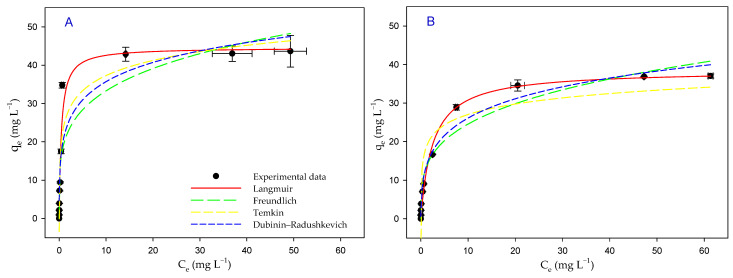
Equilibrium isotherms of TB biosorption by the microalgal biomass in the presence of light (**A**) and in the dark (**B**).

Biosorption isotherms are an excellent model to evaluate the sorption capacity of a sorbent, in this case, the microalgal biomass of *P. tricornutum*. Figure 4 represents the equilibrium data obtained for TB removal with the microalgal biomass, both in the presence of light and in the dark, together with the fit to the isotherm models, which are already indicated in Table 1. Table 4 shows all the values obtained for the parameters derived from fitting the equilibrium data to the different isotherm models, both in the presence of light and in the dark. Taking into account the *r_adj_*^2^ values obtained, the order of best to worst fit was Langmuir > Temkin > Freundlich > Dubinin–Radushkevich for the data obtained with the biomass in the presence of light, while in the dark, the order was Langmuir > Dubinin–Radushkevich > Freundlich > Temkin. As can be seen in Figure 4, the data obtained in the presence of light were different from those obtained in the dark. Thus, the values obtained at equilibrium were higher in the presence of light. In addition, the flattening of experimental values was reached with higher values of the dye concentration in the medium at equilibrium, and that flattening happened less abruptly and with lower values of *q_e_*.

Considering the data obtained with the Langmuir isotherm, the maximum sorption capacity (*q_max_*) for TB in the presence of light was 45 ± 2 mg g^−1^, while in the absence of light, this capacity was only of 39 ± 1 mg g^−1^. The *K_F_* parameter of the Freundlich isotherm showed that the biomass in the presence of light had a higher affinity for TB (20 ± 3 L mg^−1^) than in the dark (13 ± 2 L mg^−1^). The Temkin isotherm also confirmed this difference between the light and dark experiments since the constant *q_T_* was higher in the presence of light. The Dubinin–Radushkevich isotherm indicates the free energy of sorption (*E_D_*), which can be 8–16 KJ mol^−1^, indicating that the process is mainly carried out by means of chemisorption, or if it is less than 8 KJ mol^−1^, the sorption would be due to physical mechanisms [33]. Since the results were located between 8 and 16 KJ mol^−1^ in both conditions, it can be assumed that the biosorption of the TB dye was mainly carried out by means of chemisorption.

### 3.7. Effect of pH on Biosorption Efficiency

**Figure 5:**
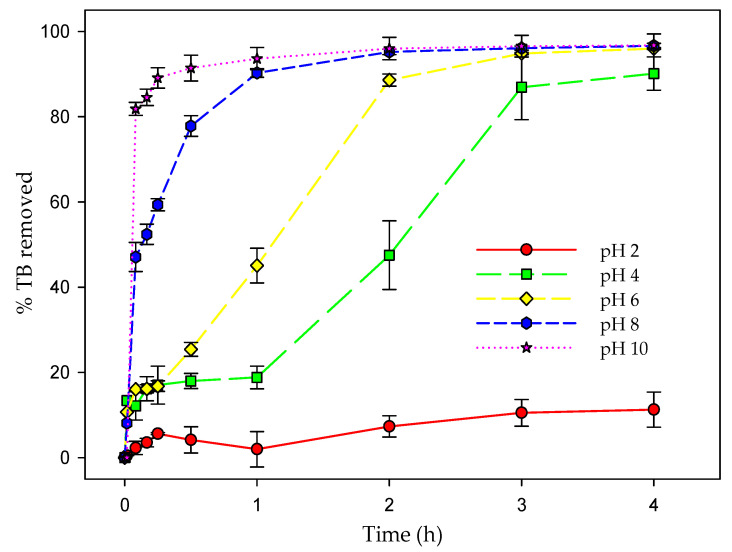
Evolution of the % of dye removed over time at the different pH values tested. The concentration of dye was 20 mg L^−1^.

For these assays, the stability of the dye in seawater at the different pH values tested and in the presence of light was first evaluated. The results obtained and analyzed by means of a two-way ANOVA, using time (initial and final) and the different pH values as influencing factors, indicated that there were no significant differences in either factor (F_1,20_ = 1.83; *p* = 0.16); therefore, the dye was stable for the duration of the process at all the pH values tested. Figure 5 shows the data obtained for the percentage of dye removal for the different pH values tested over time. The results analyzed by means of a two-way ANOVA (factors: time (initial and final), and pH value) showed that there were significant differences (F_1,20_ = 11,473.71; *p* < 0.001) in the influence of pH on the efficiency of the microalgal biomass to remove the dye. After 4 h of testing, no significant differences (*p* > 0.05) were obtained in the percentage of TB removed at pH 6, 8, and 10. However, there were significant differences (*p* < 0.05) with respect to lower pH values. At a low pH, the sorption capacity of the microalgal biomass was very low, removing only 11% after 4 h at pH 2. The removal capacity increased progressively as the pH value of the medium increased, up to pH 10 with a removal of 98%. Therefore, pH had a direct influence on the TB removal capacity of the microalgal biomass, increasing its efficiency at a high pH, and reducing its efficiency drastically at a low pH.

### 3.8. Determination of pH_ZCP_

**Figure 6:**
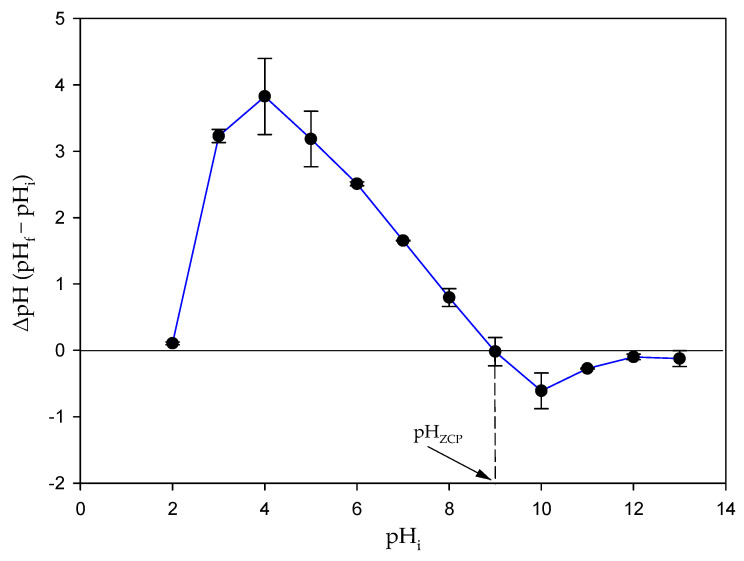
Determination of the zero charge point of the biomass of *P. tricornutum*. Values are mean ± SD (n = 3).

Figure 6 shows the evaluation of the pH of the zero charge point of the microalgal biomass. This value corresponds to the pH value where no variation occurs between the initial and final pH values, that is, where the curve cuts the line corresponding to the zero-variation value. The value obtained for the pH_ZCP_ of this biomass of *P. tricornutum* was 8.98 ± 0.21.

## 4. Discussion

Currently, dyes such as TB have a notorious presence in natural environments, causing problems due to both their direct and indirect effects on the affected environment [34]. Biosorption is considered an efficient technique for the removal of pollutants from aqueous media. However, marine environments are more neglected in terms of studies for the removal of pollutants through biosorption. For this reason, this work focused on determining the properties of the living biomass of the marine microalga *Phaeodactylum tricornutum* for the removal of TB dye from seawater using biosorption. However, it is necessary to consider that the use of seawater and illumination in the experiments carries the possibility that the dye may be unstable under these conditions, since seawater has very different characteristics from the deionized water solutions with which most biosorption experiments are carried out [35]. This instability would cause a decrease in the initial concentration of the dye, without the involvement of the biosorbent in the process, and, therefore, erroneous data would be obtained on the true properties of said biosorbent to remove the dye. The results obtained from the controls indicated that the TB dye was stable in seawater, even with illumination. Checking the stability of the sorbate in biosorption studies should be an obligatory first step for the correct characterization of the process and the biomass used. However, this procedure is unusual since it is assumed that the sorbate is stable in the test solution (usually deionized water), which may not always be correct [35].

Taking into account the results obtained, the biomass of this marine microalga was shown to have good characteristics to reduce the concentration of TB in seawater. In fact, microalgae are included in the group of so-called emerging biosorbents, given the high effectiveness of microalgal biomass in removing pollutants [36]. The characteristics of the microalgal surface facilitate the capture of pollutants. In the case of the microalga *P. tricornutum*, the FTIR spectrum [18,19,20] showed that this microalga has a wide variety of functional groups, which can allow the capture of this dye through various interactions, cationic exchange, pi interactions, electrostatic attractions, and hydrogen bonds. Thus, previous studies to characterize the biomass of this microalga indicated the presence of O-H groups corresponding to alcohols, phenols, and tricarboxylic acids, the presence of amide I and amide II groups corresponding to proteins, the presence of carbohydrates, and the presence of -−C=O groups that correspond to carboxyl groups [18,19,20]. It is known that these groups facilitate the biosorption of cationic dyes; in particular, a strong interaction has been suggested between these groups, when they carry a negative charge, and the =N^+^-(CH_3_)_2_ group of the dye [37].

Although it is more common to use dead biomass in biosorption processes, the type of biomass (live or dead) can have an influence on the efficiency of the process. For this reason, it is of interest to study the effect of the application of living biomass on biosorption processes. In the present work, living microalgal biomass was used in order to study the efficiency of TB removal. Although there are environmental factors that affect both living and dead biomass, there are factors that would only have an impact on living biomass. Thus, since the living biomass of the microalga was used in this work, illumination was studied as an important factor for the efficiency of the biosorption process using this photosynthesizing microorganism. In this way, experimental data were obtained with the living biomass both in the presence of light and in the dark. The fit of the experimental data to the kinetic models allowed us to establish that the kinetics that best fitted these data, both in light and dark conditions, were the pseudo-second order kinetics, indicating a rapid removal of TB from the medium by the microalgal biomass. This is in agreement with most biosorption studies, indicating the superiority of this model for modeling these types of processes [38]. When the rates (*K*_2_) obtained with this model for this biomass were compared with the rates of other sorbents used to remove this dye [10,16], it was observed that the removal rate with the *P. tricornutum* biomass was slower. The fact of using live cells could be related to this result. For complete removal, the dye would have to enter the cell interior, which would slow down the process. The parameters derived from these kinetics indicated that illumination was a factor that increased the efficiency of the process. Not only was the amount of dye removed higher, but the removal rate was also higher in the presence of light (Table 2 and Table 3) with higher *K*_2_ values. In addition, the data obtained from the equilibrium isotherms (Table 4) provided important insights into the process. The Langmuir isotherm confirmed that there was a higher biosorption when the microalgal biomass was in the presence of a light source. The parameters derived from the Freundlich model also provided interesting information on the properties of the biomass. The *K_F_* parameter, which indicates the affinity of the *P. tricornutum* biomass for TB, obtained a higher value in the presence of light than in the dark. Finally, the Dubinin–Radushkevich model indicated that the biosorption of the TB dye was mainly carried out by means of chemisorption, both in the presence of light and in the dark.

Therefore, the experimental results showed a higher TB removal efficiency when the microalgal biomass was in the presence of light than in the dark (Figure 2). *P. tricornutum*, as a photosynthetic microorganism, has a highly light-dependent metabolism [39]. This fact leads to the biostimulation of the microalga by light, which leads to an increase in its metabolism, favoring a higher uptake of TB from the medium, which could even be degraded by the metabolism of the microalga. The illumination makes this living biomass more active, moving it away from what would be more similar to a dead biomass. Furthermore, in the effect of microalgal biomass on TB dye removal in the presence of light, a significant interaction was obtained between the amount of dye removed and the initial dye concentration. A lower concentration up to 40 mg L^−1^ removed a higher amount of dye than expected. This interaction also occurred in the dark, but only up to a concentration of 10 mg L^−1^. This could be another argument in favor of the use of living biomass to promote the efficiency of the biosorption process. At low initial concentrations of the dye, the possible toxic effect of this compound would be lower and, therefore, the living biomass would be more active. This greater activity (or greater resistance to the possible toxicity of the dye) would occur in the presence of light, with greater removal up to a concentration of 40 mg L^−1^.

These results are important to assess the use of living biomass in biosorption processes. Living biomass can use different additional mechanisms to remove TB, such as bioaccumulation or biotransformation. In fact, there are different studies that support the use of living biomass to improve the efficiency of the biosorption process [19,25].

pH is an important factor in biosorption processes because it directly influences the charge on the sorbent surface, increasing or decreasing its affinity for pollutant uptake. In turn, some pollutants may be susceptible to having a charge depending on pH. This is the case of the TB dye whose charge varies with the pH of the medium. In fact, the results obtained in this work indicated that pH was a factor that influenced the removal efficiency of the TB dye by the biomass of this microalga (Figure 5). At low pH values, the efficiency of the biomass to remove TB was clearly lower. This efficiency increased with increasing pH. There are several reasons that may explain this result. On the one hand, this dye has two *pKa* values, which are 2.4 and 11.6 [40]. This means that at pH values lower than the first value, the dye has two positive charges, while at pH values between 2.4 and 11.6, the dye has only one positive charge, and it loses this charge at pHs higher than 11.6. Furthermore, it is necessary to take into account that the pH of the zero charge point of the microalgal biomass is 8.98 ± 0.21, which means that at pH values below this point, the charge of the microalgal biomass is positive, while at higher pH values, the charge of the biomass is negative. For these reasons, as the pH value increases, dye uptake becomes more efficient. As pH approaches the zero charge point of the microalgal biomass, the positive charge on the microalgal surface decreases, but there is also a decrease in the dye, and the electrostatic repulsion between dye and biomass becomes less noticeable. If pH continues to increase, and exceeds the zero charge point, the microalgal biomass becomes negatively charged, but the dye still retains a positive charge (as long as pH 11.6 is not exceeded), increasing the electrostatic attraction; therefore, the uptake of the dye is more effective. Taking into account this effect of pH and the interaction with functional groups explained above, electrostatic interactions seem to play an important role in the removal mechanism of this dye by the biomass of *P. tricornutum*. However, information derived from isotherm models (Dubinin–Radushkevich) indicated an additional contribution from chemisorption processes. Although it cannot be ruled out that there was a chemical reaction between the dye and the complex microalgal surface, the alternative explanation is that since it is a living system, the dye could interact with the cells through more complex processes. The dye could enter the cells and interact with intracellular components or could even be metabolized. These complex processes would raise the energy necessary for the removal of the dye, which would result in an apparent chemisorption.

Another reason to explain this higher efficiency when the pH of the medium is increased is that living microalgal biomass was used, whose pH optimum is usually between 7 and 9. As the pH of the medium moves away from these values, the living microalga loses activity, which also contributes to lower dye removal when using living biomass.

In biosorption studies, it is important to highlight the different materials or organisms that showed the best efficiency in the removal of the pollutant in question in order to optimize the process. For this purpose, it is necessary to be able to compare the results obtained with different sorbents (Table 5). However, for a correct comparison, the test conditions should be as similar as possible. In this work, the assays of microalgal biomass to remove TB were carried out with seawater. As previously indicated, biosorption assays using this type of solution are rare. In fact, no data have been found in the literature with assays of this dye in seawater, so comparing different sorbents for TB under different conditions may be of little significance. However, this information can be used as a reference, but taking into account that the assays with the materials indicated in the table were carried out with deionized water, instead of seawater, as was done in the present study. Thus, the biomass of *P. tricornutum* had a maximum removal capacity of 45 ± 2 mg g^−1^ in the presence of light, and with a high affinity for the dye, according to the value of the Freundlich *K_F_* constant. As can be seen in Table 5, this value was even higher than those obtained with other biomasses, and it should be noted that this result was obtained in a complex solution such as seawater.

As can be seen in Table 5, the nature of the studied sorbents for the removal of TB is very broad. Within this group, there are sorbents with a dye removal capacity higher (in deionized water) than that of the microalga biomass, but these sorbents can also have a series of disadvantages, such as the fact that some are more expensive, require more preparation work, or even the possible difficulty of implementing them in a more complex natural environment, such as the marine environment. However, the *P. tricornutum* microalgal biomass is a cheap, easy to use, and efficient biological material for use in the marine environment.

## 5. Conclusions

To our knowledge, the present work was the first to carry out a biosorption study of Toluidine blue dye using seawater. Against this background, this study is important to assess the effectiveness of the biosorption technique under the conditions of the marine environment, since the ultimate destination of most pollutants is to accumulate in the ocean. The search for biosorbents that are efficient in the environmental conditions of the place where these biosorbents are going to be used is crucial for the use of this technique. Thus, the living biomass of the marine microalga *Phaeodactylum tricornutum* showed good qualities to reduce the concentration of TB in seawater. The results obtained indicated that the biomass of this microalga can be a suitable biosorbent to remove the TB dye from seawater. This diatom has a wide distribution as part of the marine phytoplankton, and no environmental problem associated with the presence of this species has been described, even when it is in higher quantities than usual. Therefore, this biomass could be used in bioremediation processes in the marine environment.

## Figures and Tables

**Table 1:** Equations of the kinetic and isotherm models.

Kinetics		Isotherms	
		Langmuir [26]	
Pseudo-first order [27]		$q_{e}=\frac{\left(q_{max}K_{L}C_{e}\right)}{\left(1+K_{L}C_{e}\right)}$	(3)
$q=q_{e}\left(1-e^{-k_{1}t}\right)$	(4)	Freundlich [28]	
		qe=KFCe1n	(5)
		Temkin [29,30]	
		$q_{e}=q_{T}*\ln\left(A_{T}C_{e}\right)$	(6)
Pseudo-second order [31]		Dubinin–Radushkevich [32]	
$q=\frac{q_{e}^{2}k_{2}t}{1+q_{e}k_{2}t}$	(7)	$q_{e}=q_{max}*e^{-B_{D}(RT*ln{\left(\frac{sol}{Ce}\right))}^{2}}$	(8)
		$E_{D}=\frac{1}{\sqrt[{2}]{2B_{D}}}$	(9)
*q* (mg g^−1^) is the mass of TB biosorbed per unit of biomass over the course of time *t* (h), *k*_1_ (h^−1^) is the constant of the first order kinetic model, *q_e_* (mg g^−1^) is the mass of TB absorbed at equilibrium, and *k*_2_ (g mg^−1^ h^−1^) is the constant of the second order kinetic model.		*q_e_* (mg g^−1^) is the mass of TB biosorbed per unit of biomass at equilibrium, *q_max_* (mg g^−1^) is the maximum sorption capacity, *K_L_* (L mg^−1^) is the affinity constant of the material, *C_e_* (mg L^−1^) is the TB concentration at equilibrium, *K_F_* (L mg^−1^) is the Freundlich constant, *n* is the intensity of the Freundlich constant, *q_T_* the surface capacity for pollutant sorption per unit binding energy (mg g^−1^), *A_T_* (L mg^−1^) is the binding energy constant, *R* is the ideal gas constant (8.314 J mol^−1^ K^−1^), *T* is temperature at 291 K, *B_D_* is the free energy of sorption per mole sorbate (mol^2^ J^−2^), *sol* is the solubility of the dye, and *E_D_* is the apparent energy (KJ mol^−1^).	

**Table 2:** Parameters of kinetic models for the removal of TB by microalgal biomass under the influence of light.

Initial Concentration (mg L^−1^)	Pseudo-First Order			Pseudo-Second Order		
	q_e_ (mg g^−1^)	K_1_ (h^−1^)	r_adj_^2^	q_e_ (mg g^−1^)	K_2_ (g mg^−1^ h^−1^)	r_adj_^2^
1	2.2 ± 0.0	56 ± 8	0.9733	2.2 ± 0.0	38 ± 4	0.9948
2	5.0 ± 0.1	72 ± 15	0.9503	5.2 ± 0.1	21 ± 4	0.9813
4	9.2 ± 0.3	26 ± 7	0.9320	9.6 ± 0.2	4.2 ± 0.6	0.9889
8	17 ± 1	46 ± 10	0.9445	18 ± 0	3.5 ± 0.5	0.9894
10	22 ± 0.6	50 ± 11	0.9451	23 ± 0	3.0 ± 0.4	0.9876
20	41 ± 1	32 ± 6	0.9651	43 ± 0	1.1 ± 0.1	0.9984
40	81 ± 2	29 ± 6	0.9624	85 ± 1	0.50 ± 0.04	0.9964
60	94 ± 5	43 ± 16	0.8530	101 ± 4	0.44 ± 0.13	0.9420
80	100 ± 5	18 ± 5	0.8800	104 ± 3	0.35 ± 0.09	0.9576
100	104 ± 3	13 ± 2	0.9564	109 ± 1	0.21 ± 0.02	0.9941

**Table 3:** Parameters of kinetic models for the removal of TB by microalgal biomass in the absence of light.

Initial Concentration (mg L^−1^)	Pseudo-First Order			Pseudo-Second Order		
	q_e_ (mg g^−1^)	K_1_ (h^−1^)	r_adj_^2^	q_e_ (mg g^−1^)	K_2_ (g mg^−1^ h^−1^)	r_adj_^2^
1	2.1 ± 0.1	60 ± 18	0.9088	2.2 ± 0.1	32 ± 9	0.9616
2	4.9 ± 0.2	55 ± 156	0.9149	5.1 ± 0.1	13 ± 3	0.9673
4	9.0 ± 0.3	25 ± 7	0.9232	9.5 ± 0.2	4.1 ± 0.6	0.9853
8	17 ± 0	39 ± 7	0.9689	18 ± 0	3.3 ± 0.3	0.9962
10	22 ± 0	39 ± 6	0.9755	23 ± 0	2.5 ± 0.1	0.9990
20	40 ± 1	9.2 ± 1.6	0.9426	42 ± 1	0.39 ± 0.06	0.9856
40	69 ± 3	15 ± 3	0.9299	72 ± 1	0.39 ± 0.06	0.9841
60	83 ± 2	14 ± 2	0.9792	88 ± 1	0.27 ± 0.01	0.9989
80	90 ± 2	14 ± 1	0.9867	94 ± 1	0.26 ± 0.03	0.9921
100	88 ± 3	6.4 ± 0.8	0.9685	94 ± 1	0.10 ± 0.01	0.9952

**Table 4:** Parameters of isotherm models for TB biosorption by the microalgal biomass in the presence of light and in darkness.

Isotherm	Parameters	Light	Darkness
Langmuir	q_max_ (mg g^−1^)	45 ± 2	39 ± 1
	K_L_ (L mg^−1^)	2.1 ± 0.4	0.4 ± 0.1
	r_adj_^2^	0.9634	0.9880
Freundlich	K_F_ (L mg^−1^)	20 ± 3	13 ± 1
	1/n	4.3 ± 0.9	3.6 ± 0.4
	r_adj_^2^	0.8377	0.9581
Temkin	q_T_ (mg g^−1^)	5.7 ± 0.6	3.9 ± 0.6
	A_T_ (L mg^−1^)	70 ± 37	101 ± 82
	r_adj_^2^	0.8969	0.8429
Dubinin– Radushkevich	q_max_ (mg g^−1^)	79 ± 13	75 ± 7
	B_D_ (mol^2^ J^−2^)	2.1 × 10^−9^ ± 4.1 × 10^−10^	2.9 × 10^−9^ ± 2.8 × 10^−10^
	E_D_ (KJ mol^−1^)	15 ± 2	13 ± 1
	r_adj_^2^	0.8571	0.9714

**Table 5:** Different sorbents used in the removal of Toluidine blue from aqueous solution.

Sorbents	q_max_ ^†^ (mg g^−1^)	K_F_ ^††^ (L mg^−1^)	Contact Time (h)	pH	Sorbent Concentration (g L^−1^)	TB Concentration (mg L^−1^)	Reference
**Graphene oxide/bentonite**	459	242	2	8	0.5	200–800	[41]
**Orange peel waste**	313	3.3	0.5	10	3	20–160	[42]
**Neem leaf powder**	187	4.0	8	7	3	0.0001–0.0009	[43]
***Phaeodactylum tricornutum***	45	20	4	8	0.4	1–100	This work (seawater)
**Gypsum**	28	6	1	6.5	1	0.03–0.3	[3]
***Lemna minor***	27	7.2	24	---	---	5–40	[16]

^†^ Parameter obtained from a Langmuir isotherm. ^††^ Freundlich constant.

## Data Availability

The datasets generated during and/or analyzed during the current study are available from the corresponding author on reasonable request.

## References

[1] Robinson T., McMullan G., Marchant R., Nigam P. (2001). Remediation of dyes in textile effluent: A critical review on current treatment technologies with a proposed alternative. Bioresour. Technol..

[2] Kant R. (2011). Textile dyeing industry an environmental hazard. J. Nat. Sci..

[3] Rauf M.A., Qadri S.M., Ashraf S., Al-Mansoori K.M. (2009). Adsorption studies of Toluidine Blue from aqueous solutions onto gypsum. J. Chem. Eng..

[4] Khataee A.R., Movafeghi A., Torbati S., Salehi Lisar S.Y., Zarei M. (2012). Phytoremediation potential of duckweed (*Lemna minor* L.) in degradation of C.I. Acid Blue 92: Artificial neural network modeling. Ecotoxicol. Environ. Saf..

[5] Jalilian N., Najafpour G.D., Khajouei M. (2020). Macro and micro algae in pollution control and biofuel production—A review. Chem. Bio Eng..

[6] Poots V., Mckay G., Healy J. (1976). The removal of acid dye from effluent using natural adsorbents—I peat. Water Res..

[7] Nawar S.S., Doma H.S. (1989). Removal of dyes from effluents using low-cost agricultural by-products. Sci. Total Environ..

[8] Martínez-Jerónimo F., Cruz-Cisneros J.L., García-Hernández L. (2008). A comparison of the response of *Simocephalus mixtus* (Cladocera) and *Daphnia magna* to contaminated freshwater sediments. Ecotoxicol. Environ. Saf..

[9] Chekroun K.B., Sánchez E., Baghour M. (2014). The role of algae in bioremediation of organic pollutants. J. Pub. Environ. Heatlh.

[10] Alpat S.K., Özbayrak Ö., Alpat Ş., Akçay H. (2008). The adsorption kinetics and removal of cationic dye, Toluidine Blue O, from aqueous solution with Turkish zeolite. J. Hazard. Mater..

[11] Salim H.A.M., Idrees S.A., Rashid R.A., Mohammed A.A., Simo S.M., Khalo I.S. Photo-catalytic degradation of Toluidine Blue Dye in Aqueous Medium Under Fluorescent Light. Proceedings of the International Conference on Advanced Science and Engineering.

[12] Venkatesan D., Umasankar S., Mangesh V.L., Krishnan P.S., Tamizhdurai P., Kumaran R., Baskaralingam P. (2023). Removal of Toluidine blue in water using green synthesized nanomaterials. S. Afr. J. Chem. Eng..

[13] Wang T., Li B., Wu L., Yin Y., Jiang B., Lou J. (2019). Enhanced performance of TiO_2_/reduced graphene oxide doped by rare-earth ions for degrading phenol in seawater excited by weak visible light. Adv. Powder Technol..

[14] Xu H., Hao Z., Feng W., Wang T., Li Y. (2021). Mechanism of Photodegradation of Organic Pollutants in Seawater by TiO_2_-based Photocatalysts and Improvement in Their Performance. ACS Omega.

[15] Mustafa S., Bhatti H.N., Maqbool M., Iqbal M. (2021). Microalgae biosorption, bioaccumulation and biodegradation efficiency for the remediation of wastewater and carbon dioxide mitigation: Prospects, challenges and opportunities. J. Water Process. Eng..

[16] Neag E., Malschi D., Măicăneanu A. (2018). Isotherm and kinetic modelling of Toluidine Blue (TB) removal from aqueous solution using *Lemna minor*. Int. J. Phytoremediation.

[17] Sangwan S., Dukare A. (2018). Microbe-mediated bioremediation: An eco-friendly sustainable approach for environmental clean-up. Advances in Soil Microbiology: Recent Trends and Future Prospects.

[18] Santaeufemia S., Abalde J., Torres E. (2019). Eco-friendly rapid removal of triclosan from seawater using biomass of a microalgal species: Kinetic and equilibrium studies. J. Hazard. Mater..

[19] Santaeufemia S., Abalde J., Torres E. (2021). Efficient removal of dyes from seawater using as biosorbent the dead and living biomass of the microalga *Phaeodactylum tricornutum*: Equilibrium and kinetics studies. J. Appl. Phycol..

[20] Santaeufemia S., Torres E., Abalde J. (2016). Bioremediation of oxytetracycline in seawater by living and dead biomass of the microalga *Phaeodactylum tricornutum*. J. Hazard. Mater..

[21] Mohd Udaiyappan A.F., Abu Hasan H., Takriff M.S., Sheikh Abdullah S.R. (2017). A review of the potentials, challenges and current status of microalgae biomass applications in industrial wastewater treatment. J. Water Process. Eng..

[22] Kurano N., Ikemoto H., Miyashita H., Hasegawa T., Hata H., Miyachi S. (1995). Fixation and utilization of carbon dioxide by microalgal photosynthesis. Energy Conv. Manag..

[23] Kaithwas A., Prasad M., Kulshreshtha A., Verma S. (2012). Industrial wastes derived solid adsorbents for CO_2_ capture: A mini review. Chem. Eng. Res. Des..

[24] Singh J., Dhar D.W. (2019). Overview of carbon capture technology: Microalgal biorefinery concept and state-of-the-art. Front. Mar. Sci..

[25] Santaeufemia S., Torres E., Abalde J. (2018). Biosorption of ibuprofen from aqueous solution using living and dead biomass of the microalga *Phaeodactylum tricornutum*. J. Appl. Phycol..

[26] Langmuir I. (1918). The adsorption of gases on plane surfaces of glass, mica and platinum. J. Am. Chem. Soc..

[27] Lagergren S.K. (1898). About the theory of so-called adsorption of soluble substances. Sven. Vetenskapsakad. Handingarl.

[28] Freundlich H. (1906). Over the adsorption in solution. J. Phys. Chem..

[29] Temkin M., Pyzhev V. (1940). Recent modifications to Langmuir isotherms. Acta. Physiochim. URSS.

[30] Chu K.H. (2021). Revisiting the Temkin Isotherm: Dimensional Inconsistency and Approximate Forms. Ind. Eng. Chem. Res..

[31] Blanchard G., Maunaye M., Martin G. (1984). Removal of heavy metals from waters by means of natural zeolites. Water Res..

[32] Dubinin M., Radushkevich L. (1947). The equation of the characteristic curve of activated charcoal. Proc. Acad. Sci. Phys. Chem. Sect. USSR.

[33] Quili H., Zhenya Z. (2019). Application of Dubinin-Radushkevich isotherm model at the solid/solution interface: A theoretical analysis. J. Mol. Liq..

[34] Vacchi F.I., Vendemiatti J.A.d.S., da Silva B.F., Zanoni M.V.B., Umbuzeiro G.d.A. (2017). Quantifying the contribution of dyes to the mutagenicity of waters under the influence of textile activities. Sci. Total Environ..

[35] González V., Abalde J., Torres E. (2024). Discoloration and biosorption of brilliant green dye in seawater using living biomass of the microalga *Phaeodactylum tricornutum*. J. Appl. Phycol..

[36] Xiong J.-Q., Kurade M.B., Jeon B.-H. (2018). Can microalgae remove pharmaceutical contaminants from water?. Trends Biotechnol..

[37] Qingfeng W., Kristen C., Qi C., Xisen W., Zhaohui L. (2021). Interactions between cationic dye toluidine blue and fibrous clay minerals. Crystals.

[38] Tan K.L., Hameed B.H. (2017). Insight into the adsorption kinetics models for the removal of contaminants from aqueous solutions. J. Taiwan Inst. Chem. Eng..

[39] Siaut M., Heijde M., Mangogna M., Montsant A., Coesel S., Allen A., Manfredonia A., Falciatore A., Bowler C. (2007). Molecular toolbox for studying diatom biology in *Phaeodactylum tricornutum*. Gene.

[40] Salim H.A.M., Salih S.A.M. (2015). Photodegradation study of Toluidine Blue dye in aqueous solution using magnesium oxide as a photocatalyst. Int. J. Chem..

[41] Xu W., Chen Y., Zhang W., Li B. (2019). Fabrication of graphene oxide/bentonite composites with excellent adsorption performances for toluidine blue removal from aqueous solution. Adv. Powder Technol..

[42] Lafi R., Rezma S., Hafiane A. (2015). Removal of toluidine blue from aqueous solution using orange peel waste (OPW). Desalin. Water Treat..

[43] Patel H., Vashi R. (2010). A study on removal of Toluidine blue dye from aqueous solution by adsorption onto Neem leaf powder. World Acad. Sci. Eng. Technol..

